# Efficiency of Artificial Intelligence in Three-Dimensional Reconstruction of Medical Imaging

**DOI:** 10.7759/cureus.96580

**Published:** 2025-11-11

**Authors:** Nikhita, Dhyaan Bannur, Maria Gabriela Cerdas, Arisha Zahid Saeed, Bashir Imam, Ravtej Singh Thandi, Anusha H C, Preethi Reddy, Ramsha Ali

**Affiliations:** 1 Acute Medicine, Good Hope Hospital, Birmingham, GBR; 2 Basic Sciences, University of Texas at Dallas, Richardson, USA; 3 Medicine, Universidad de Ciencias Médicas, San Jose, CRI; 4 Medicine, Gulf Medical University, Ajman, ARE; 5 Pediatrics, University of Pittsburgh Medical Center, Coudersport, USA; 6 Medicine, Wenzhou Medical University, Wenzhou, CHN; 7 Department of Radiology, Kaloji Narayana Rao University of Health Sciences, Warangal, IND; 8 Medicine and Surgery, Peoples University of Medical and Health Sciences for Women, Hyderabad, PAK

**Keywords:** 3d reconstruction, ai in medical imaging, artificial intelligence (ai), ct and mri, image segmentation, oncology imaging, radiology, technology in healthcare

## Abstract

Three-dimensional (3D) reconstruction is necessary for visualizing complex anatomy and supporting clinical decision-making in radiology. However, traditional techniques often struggle with limitations in scalability, speed, and reproducibility. The recent emergence of artificial intelligence (AI) has enabled a new generation of reconstruction tools that offer greater automation and new clinical capabilities. Motivated by the demand for efficient imaging, researchers have applied deep learning to overcome longstanding barriers, with impacts spanning diagnosis, surgical planning, and disease monitoring. This review included peer-reviewed studies published within the last 10 years, focusing exclusively on adult human imaging published in the English language, as anatomical development, imaging protocols, and clinical decision pathways differ significantly in pediatric populations. While this improved applicability to adult radiology, it limited insight into emerging pediatric and preclinical research. Searches focused on AI-driven 3D reconstruction across different radiologic modalities. Articles were selected based on the following predefined inclusion criteria: adult human participants, AI-based 3D reconstruction, clinical validation, and relevance to radiologic practice. Studies including pediatric or animal subjects, preclinical-only experimentation, non-English-language text, or without applied clinical evaluation were excluded. Our results showed that AI has improved the accuracy, speed, and clinical utility of 3D reconstruction in multiple specialties. Deep learning models such as U-Net, V-Net, DenseVNet, and generative adversarial networks have achieved high segmentation accuracy, often reporting Dice scores >0.90. These models have also been used for tumor detection, surgical planning, and reducing radiation exposure. However, challenges such as high computational requirements, lack of standardized datasets, limited real-world validation, and ethical concerns remain. In conclusion, 3D reconstruction is transforming radiology with more accurate patient-specific images for improved clinical decision-making. While it is rapidly being integrated into practice, these technologies still have limitations that need to be addressed. Nonetheless, with improved and ongoing innovation, AI has the potential to become a catalyst for precise imaging and patient care.

## Introduction and background

Three-dimensional (3D) reconstruction has become a critical component of diagnostic radiology, enabling volumetric visualization of complex anatomical structures for clinical decision-making and surgical guidance. Conventional reconstruction pipelines rely heavily on manual segmentation and radiologist expertise, which introduces operator-dependent variability, contributes to interobserver inconsistency, and increases interpretation time, particularly in subspecialties that require high spatial precision.

Artificial intelligence (AI) has driven a major evolution in imaging by enhancing the accuracy, speed, and scalability of automated 3D reconstruction techniques. Historically, clinicians relied on manual segmentation of CT, MRI, and ultrasound datasets, which required extensive expertise and was highly dependent on operator skill, experience, and availability. These manual approaches are inherently prone to variability between users, increased error rates, and longer interpretation times, especially in complex anatomical cases that require precise delineation of fine structures.

However, the recent advances in deep learning, machine intelligence, and computer vision have revolutionized these procedures. 3D reconstruction has become more accurate, efficient, and accessible than ever. AI-based algorithms can now automate segmentation and highlight possible abnormalities that might otherwise go unnoticed. The higher precision benefits radiologists in clinical workflows, whether for cancer staging or surgery planning, especially for adult patients. As these tools improve, they are reshaping both the patient experience and the daily operations of imaging, making safer and less invasive procedures possible. For example, various U-Net-based models have been successful in accurately segmenting atria in MRIs during preoperative assessments, leading to less time being spent on manual reconstruction itself.

Across specialties, AI-enhanced segmentation has demonstrated measurable advantages over manual reconstruction. Several recent AI validation studies report reductions in interpretation time by 25-60%, increases in lesion detection sensitivity by 10-20%, and Dice similarity coefficients exceeding 0.90, frequently matching or surpassing expert-level agreement. These improvements highlight the clinical performance gains that justify continued investigation and adoption of AI-driven reconstruction methods.

How does AI improve the quality and precision of 3D reconstruction in medical imaging, and what are the implications for patient care? What are the key enablers and challenges in integrating this AI-driven 3D reconstruction into existing healthcare systems, and how can AI be utilized to overcome current limitations in traditional digital reconstruction methods?

AI-based 3D reconstruction is transforming radiology by overcoming important limitations of traditional methods, which are time-consuming, sensitive to noise, and require large amounts of manual data processing. With AI, it is possible to reconstruct high-resolution 3D images from low-quality data [1]. Recent studies have demonstrated promising advances: for instance, Dai et al. improved ultrasound resolution using a self-supervised AI method without manual segmentation, while Choi et al. enhanced breast lesion visibility in tomosynthesis with a deblurring algorithm, both improving image quality for better diagnosis [1,2].

The application of this technology extends beyond diagnostic imaging into surgical planning and intervention. Studies by Meng et al. and Minnema et al. showed that it can improve preoperative planning by enhancing visualization in knee arthroplasty and maxillofacial surgery, respectively [3,4]. Overall, many studies suggest that this approach can increase imaging accuracy, reduce radiation dose, improve efficiency, and, ultimately, benefit clinical decision-making and patient outcomes.

This review focuses on the recent development of AI-based 3D reconstruction in various imaging modalities and its applications in the fields of oncology, orthopedics, cardiology, and surgery. It also covers algorithmic innovations, their current real-world applications, remaining challenges, and potential future roles in medical practice. This analysis is limited to studies published in the last 10 years that involve adult participants (18 years and older), with all studies focusing exclusively on human subjects. Articles published in languages other than English, research involving pediatric or pregnant populations, and investigations focusing solely on theoretical or preclinical animal studies were excluded.

Recent progress in AI-driven 3D reconstruction techniques has shown noticeable improvements in radiology practices, especially in adult clinical practices. Detailed anatomical models have been created to help radiologists detect features in complex cases and diseases [5-7]. This new automation in AI would significantly reduce the processing time for imaging and reduce the variability present between users, which would overall allow faster and more consistent results [8,9]. Moreover, the AI-driven techniques are making precise progress in preoperative and interventional planning. Surgeons can effectively plan procedures quickly with reliable 3D models, leading to better patient outcomes [10,11]. The adoption of these techniques is growing with increased approval in many hospitals, and clinical benefits have been observed in many studies [12,13]. Despite the many advantages present in preoperative planning, there is a lack of reinforced studies in the clinical application of these tools. Some of the ongoing challenges would include ensuring that the AI models work well across many different patient types, establishing more transparent clinical validation, and easing integration into busy clinical settings [5,14].

## Review

Evolution of AI-based 3D reconstruction in adult radiology

The progress of 3D reconstruction in radiology began with the transition from two-dimensional (2D) to 3D imaging. Breakthroughs started with the development of CT in the early 1970s, which propelled radiology into three dimensions, combining the rotation of X-ray sources with computational algorithms to reconstruct volumetric data from stacked 2D images [15]. Along with that, MRI soon allowed exceptionally detailed visualization of soft tissue anatomy without ionizing radiation, and the later rise of digital radiography and picture archiving and communication systems, making vast imaging datasets accessible for computational analysis [16].

In the 2000s, increased computational resources enabled model-based iterative reconstruction, which significantly improved image quality and allowed substantial dose reduction in clinical imaging. However, its widespread adoption was limited by long processing times and unfamiliar noise textures that some radiologists found difficult to interpret [5,15].

Soon, a significant leap in technology occurred with the introduction of AI and deep learning into radiological image reconstruction. Early neural networks, such as the convolutional neural network (CNN), laid the foundation for models that could learn directly from imaging data and optimize features automatically [17]. The dramatic increase in medical imaging data and advances in hardware, such as graphical processing units, enabled deep learning to surpass earlier statistical methods in reconstructing and segmenting complex anatomical structures [5,15,18].

Significant milestones would include the deployment of 3D U-Net architectures for detailed, automated organ segmentation, achieving high accuracy and reproducibility in musculoskeletal, cardiac, and neurological MRI [6,18]. The introduction of deep learning-based image denoising, artifact reduction, and super-resolution methods has enabled high-quality reconstructions from noisy, sparse, or low-dose scans, which improves patient safety and allows for use in populations unable to tolerate prolonged or high-dose protocols [19]. Explicit modeling frameworks, such as point-based and volume-based networks for bone and soft tissue, and implicit networks, including neural radiance fields, can reconstruct subtle anatomical structures for patient-specific or population-based mapping [18,20]. AI tools now enable rapid, almost real-time 3D modelling for procedural planning in thoracic, cardiac, and neuroimaging surgery, reducing planning times from hours to minutes and empowering non-experts to complete complex reconstructions with high accuracy [11,18].

**Figure 1:**
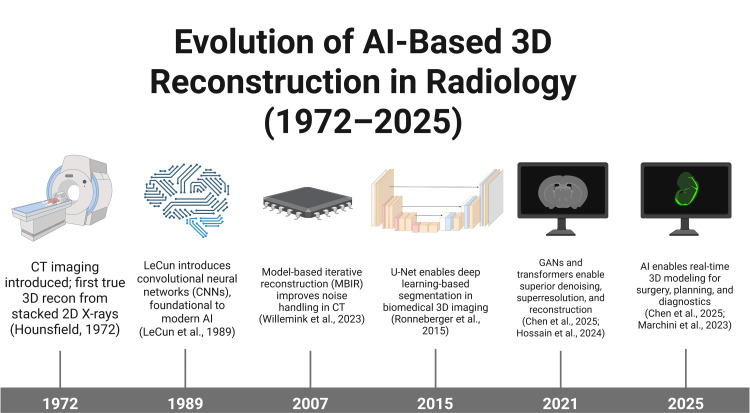
Historical timeline of technological milestones in AI-driven 3D reconstruction for radiology. This figure was created using the software BioRender by the authors. AI = artificial intelligence; 2D = two-dimensional; 3D = three-dimensional

Finally, the emergence of open-access imaging datasets and standardized evaluation metrics has accelerated benchmarking, validation, and regulatory approval for clinical use [5,16]. Today, AI-driven 3D reconstruction is not only a focus of ongoing research but also an established clinical tool in modern radiology and surgical practices worldwide (Figure 1). These advances are fueled by the rapid growth of annotated datasets, increased hardware acceleration, growing demands for dose efficiency and speed, and powerful AI architectures [16,18].

Human versus AI accuracy in 3D reconstruction

Example 1: Lung and Thoracic Imaging - Surgical Planning

**Figure 2:**
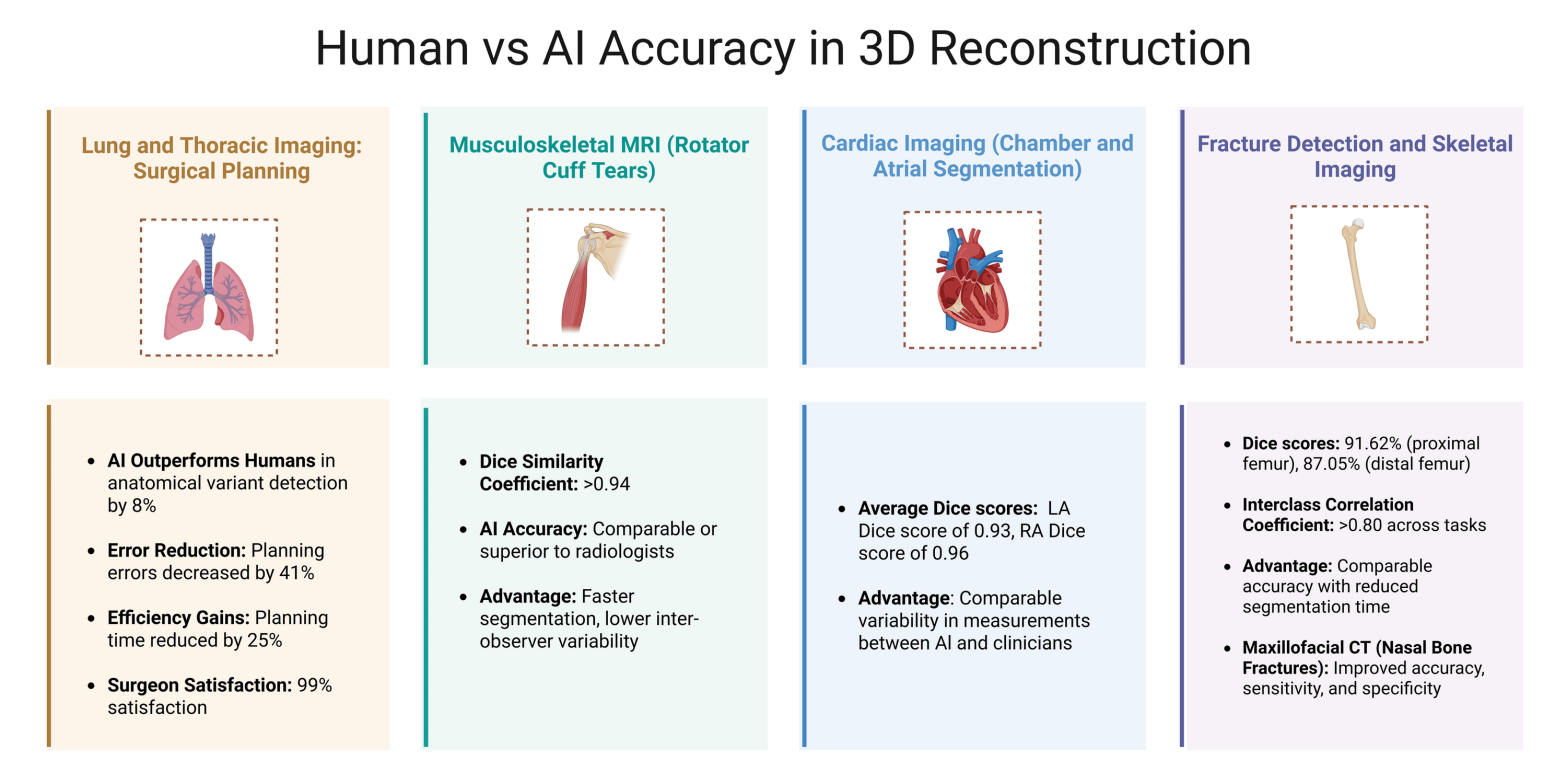
Comparative accuracy of AI and human experts in 3D anatomical reconstruction. This figure was created using the software BioRender by the authors. AI = artificial intelligence; 3D = three-dimensional

AI systems applied to lung surgery planning are now comparable to human experts in anatomical assessment and reconstruction accuracy (Figure 2). In a large multicenter, multi-reader study, an AI-powered tool for reconstructing pulmonary vessels and bronchi not only increased the identification of anatomical variants by 8% over human performance but also decreased planning errors by 41% and reduced planning time by 25%. Surgeon satisfaction with AI-assisted results approached 99%, highlighting both enhanced accuracy and tangible workflow improvements [18].

Example 2: Musculoskeletal MRI - Rotator Cuff Tears

In musculoskeletal imaging, a 3D U-Net deep learning model for rotator cuff segmentation was compared against experienced radiologists. The AI model achieved Dice similarity scores exceeding 0.94, surpassing expert consistency while delivering faster and more reproducible results (Figure 2). These improvements may enhance diagnostic confidence and reduce the need for secondary reviews. Similar AI-driven gains have been observed in broader orthopedic and soft tissue segmentation, where automation helps reduce inter-observer variability and improves reliability in ambiguous or subtle cases [21].

Example 3: Cardiac Imaging - Whole Heart and Atrial Segmentation

A fully neural network-based method was trained on a large-scale dataset including 4,875 subjects and 93,500 pixel-wise annotated images to automatically analyze cardiac magnetic resonance (CMR) images. Previously, this process was performed manually by clinicians, which often led to subjective errors and was inefficient. This method was tested via segmentation of all four heart chambers on CMR images. On an image test set of 600 subjects, the mean absolute difference for left ventricular end diastolic volume, left ventricular end systolic volume, right ventricular end diastolic volume, and left ventricular end systolic volume between manual and automated measurements displayed results with comparable variability to that between clinicians. The same conclusion was reached with the Dice metrics for the atria, which averaged 0.93 for the left atrial cavity and 0.96 for the right atrial cavity [22]. These findings highlight that AI-driven methods are incredibly efficient in achieving a level of analysis on par with clinical experts (Figure 2).

Example 4: Fracture Detection and Skeletal Imaging

A retrospective study analyzed the difference between the semantic segmentation of fracture fragments completed by humans versus a V-Net AI tool known as intertrochanteric femoral fracture CT (IFFCT). The study concluded that the AI tool displayed comparable results to human experts; however, it had significantly reduced segmentation time. Average Dice scores of IFFCT were 91.62% for the proximal femur and 87.05% for the distal femur. IFFCT results were consistently on par with those of humans, as intra-class correlation coefficients exceeded 0.80 for all regions, indicating high reliability [23].

Another study on nasal bone fractures and maxillofacial CT utilized a sample of 82 patients and determined that the AI tool improved the accuracy, sensitivity, and specificity of identification of nasal fractures. It was also emphasized that the AI tool was especially helpful for junior residents. Results indicated notable improvement when utilizing the deep learning model, as all differences were significant (p < 0.05) and higher than that of manual detection [24].

Current hospitals and healthcare systems using AI in 3D reconstruction

Over the past decades, AI in 3D reconstruction has made immense progress and gained success in practical applications in healthcare worldwide. It is now integrated into clinical workflows, especially in tertiary and research-oriented hospitals. These systems have enabled faster image processing, improved anatomical modelling, and enhanced support for surgical planning.

One notable example is the Mayo Clinic, where AI is very well integrated in cardiac and oncologic imaging pipelines. Their advanced imaging center uses CNNs to reconstruct volumetric models of vascular structures and tumors from CT and MRI data, assisting surgeons in preoperative planning and radiation therapy mapping. The AI integration not only speeds up the segmentation process but also reduces variability between operators, contributing to more standardized care [25].

Another hospital using AI to assist with 3D reconstruction is Massachusetts General Hospital. They integrated AI in 3D reconstruction in thoracic imaging, which has been helpful to radiologists for generating accurate thoracic models using AI-driven segmentation. This helps with more accurate access to tumor proximity to vessels or other major anatomical parts, improving surgical decision-making in cases of minimally invasive resections. These reconstructions have been a vital part of multidisciplinary case discussions and surgical rehearsals [26].

University College London Hospital in the United Kingdom has also embedded AI-based 3D brain mapping tools in its healthcare system for patients undergoing neurosurgical interventions. These tools use deep learning algorithms to segment and reconstruct structures from MRI scans, providing neurosurgeons with precise models to navigate tumor resections or vascular anomalies. These integrations have further enhanced intraoperative accuracy, especially in cases involving eloquent cortex [27].

Some institutes are also using AI 3D reconstruction for orthopedic applications. One such example is Singapore General Hospital, where AI 3D reconstruction is being used for creating models of the spine and pelvis from standard X-rays. These applications are helpful in planning complex spine surgeries while reducing the need for CT imaging and minimizing radiation exposure [28].

As AI-driven 3D reconstruction becomes more widely adopted in radiology, regulatory and ethical frameworks are essential for guiding responsible clinical integration. Key priorities include protecting patient privacy and preventing unauthorized reidentification of imaging data, while also addressing cybersecurity threats associated with cloud-based reconstruction workflows and third-party software integration. Furthermore, standardized oversight is necessary to ensure algorithmic transparency, equitable patient access, and accountability across healthcare environments. Despite progress in establishing safeguards, variability in implementation and enforcement continues to limit harmonized adoption, highlighting the need for coordinated governance and multidisciplinary collaboration.

Future research should prioritize prospective, multicenter clinical trials directly comparing AI-driven 3D reconstruction with standard-of-care planning across specific specialties. Study designs incorporating patient-specific modeling, intraoperative validation, and outcome-based metrics will help define where AI provides the most value in personalized medicine. Additionally, expanded work in secure model deployment and explainable AI is needed to support regulatory compliance and clinical trust. Mixed-method designs, including clinician and patient perspectives, will be critical for sustainable real-world integration.

In this review, “high-quality datasets” refer to imaging collections with standardized acquisition protocols, reliable expert annotations, and diverse patient representation to support robust model development. Ongoing multi-institution efforts aim to improve data availability and interoperability while maintaining privacy protections and institutional control.

**Figure 3:**
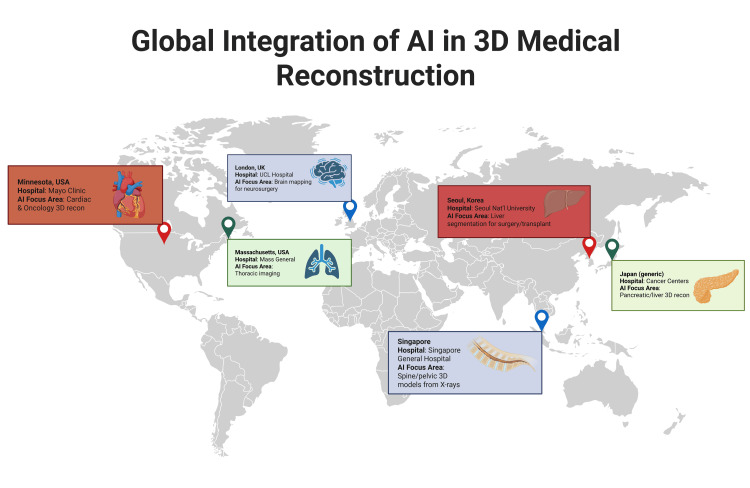
Global integration of AI-driven 3D reconstruction in clinical practice. This figure was created using the software BioRender by the authors. AI = artificial intelligence; 3D = three-dimensional

Overall, AI-driven 3D reconstruction is no longer just a theoretical tool; it has successfully been integrated in many hospital facilities around the globe, actively transforming the healthcare field (Figure 3). From preoperative planning to real-time surgical navigation, these systems are improving efficiency, accuracy, and personalization of care.

Despite strong technical performance, integrating AI-driven 3D reconstruction into routine practice requires structured clinician training, adequate workflow adaptation, and interdisciplinary support. Different specialties have variable software familiarity, imaging acquisition protocols, and surgical planning requirements, which may affect technology adoption and real-world impact. Therefore, implementation frameworks and education strategies are essential to ensure effective clinical integration.

AI-driven methods for organ segmentation based on MRI and CT imaging data

Over recent years, advancements in AI have radically improved organ segmentation in MRI, making it faster, more accurate, and less labor-intensive. Several deep learning models and methods now enable precise illustration of anatomical structures necessary for diagnosis, surgical planning, and patient management.

One such technique is DenseVNet, a high-capacity CNN that segments 13 knee structures, including cartilage, ligaments, vessels, and nerves, from 3D MRI scans. This powerful system has demonstrated accurate results; for example, in recent studies, it achieved Dice scores up to 0.998 for muscle and 0.973 for ligaments. Such performance would translate into reliable mapping of both healthy anatomy and pathologies such as anterior cruciate ligament ruptures, with significant time savings in preoperative assessment [29].

Another prominent approach employs graph neural networks for spine imaging. This model performs automated 3D segmentation and grading of lumbar intervertebral discs from sagittal MRI, providing objective Pfirrman classifications for disc degeneration. Studies show that this method achieves an 88.1% mean accuracy, effectively replicating expert clinical evaluations and improving workflow efficiency by reducing time spent on manual review [30].

Alongside MRI, CT datasets have been utilized in various AI-driven segmentation techniques. Studies show how U-Net-based deep learning models are effective on MRI and CT imaging data. Such models are incredibly adaptable and versatile as they can be used on multiple organs, for example, uterine fibroids (MRI), knee joint arthroplasty (CT), upper urinary tract (CT), and so on [6,31,32].

V-Net-based deep learning models are specifically designed for 3D segmentation and visualization of 3D volumes. These models show strong performance in both MRI and CT contexts. Adding to this, a study on femoral fractures confirms with Dice scores in IFFCT being up to 91.62% in proximal femur fractures. Thus, proving that these techniques can be effectively applied to both MRI and CT datasets [23,33].

CNNs represent a large category of AI-driven approaches in both CT and MRI image reconstruction, bone segmentation, and surgical planning. Despite being initially designed for 2D imaging, recent studies discuss the application to 3D segmentation. A 2020 study on lung nodule segmentation outlined how a multi-view secondary input residual convolutional neural network (MV-SIR-CNN) had excellent results when compared with existing CNN models, achieving a Dice coefficient of 0.926. These findings place it on par with U-Net models in terms of segmentation accuracy, reinforcing its usefulness in clinical settings [4,13,34].

In addition to these methods, dual-energy CT clustering (DECT-CLUST) is an effective unsupervised clustering approach based on dual-energy CT data, which displays strong spatial and spectral tumor delineation without manual training. Hence, being focused solely on CT data, although it can be used in conjunction with MRI. One study demonstrated that this technique could improve automated tumor detection and segmentation independent of large datasets when tested on head and neck squamous cell carcinoma, highlighting the possibility of enhancing diagnostic accuracy alongside supporting treatment planning by reducing inter-observer variability in clinical applications of radiology [35].

Together, these tools are revolutionizing how clinicians interpret MRI and CT data, offering speed, reliability, and reproducibility that manual methods cannot match (Table 1).

**Table 1:** AI-based 3D segmentation techniques in MRI versus CT. AI = artificial intelligence; 3D = three-dimensional; CNN = convolutional neural network; MV-SIR-CNN = multi-view secondary input residual convolutional neural network; DECT-CLUST = dual-energy CT clustering; HNSCC = head and neck squamous cell carcinoma

Modality	AI method	Use case/Target area	Model type	Performance (Dice/accuracy)	Notes	Reference
MRI	DenseVNet	Knee (cartilage, ligaments, vessels, nerves)	CNN	0.998 (muscle), 0.973 (ligaments)	High-precision preoperative mapping	[6]
MRI	GNN	Spine discs (Pfirrmann grading)	Graph neural network	88.1% mean accuracy	Objective disc degeneration scoring	[30]
MRI	3D U-Net	Cardiac atria, rotator cuff tears	3D CNN (U-Net)	>90% Dice	Multi-view support, fast segmentation	[22,31]
MRI	V-Net	Proximal femoral fractures	3D CNN (V-Net)	91.62% Dice	Tailored for volumetric segmentation	[23,32]
CT	U-Net	Upper urinary tract, knee arthroplasty, fibroids	CNN (U-Net-based)	Varies by organ (all high performing)	Versatile across multiple organs	[29,31]
CT	MV-SIR-CNN	Lung nodule segmentation	Multi-view CNN	0.926 Dice	Competitive with U-Net models	[34]
CT	DECT-CLUST	Tumor delineation (e.g., HNSCC)	Clustering (unsupervised)	Not reported; high spatial accuracy	Requires no manual labels; CT-specific	[35]

Clinical applications across specialties

Multiple clinical specialties are now implementing AI for 3D reconstruction to boost anatomical visualization and improve both surgery planning and diagnostic precision.

Musculoskeletal Imaging

AI applications have shown great clinical results, especially in orthopedics. This specialty has started to integrate deep learning models to improve diagnostic accuracy. One study demonstrated that a 3D U-Net CNN for the detection of rotator cuff tears achieved a Dice coefficient score of 94.3%, alongside a high sensitivity of 97.1% and specificity of 95.0% [6].

Orthopedic surgical planning is another domain where AI-enhanced 3D reconstruction methods have shown improvements. In hip arthroplasty, the use of AI-based 3D planning improved prosthetic sizing accuracy and reduced operation time while minimizing surgical blood loss and fluoroscopy exposure [36]. Similarly, an augmented reality system was used to guide tibial fracture surgery, enabling surgeons to view fracture fragments and implant positions in real-time 3D without intraoperative X-rays, thereby reducing patient radiation exposure [37]. In addition, an automatic 3D pelvimetry framework applying deep learning algorithms to CT images demonstrated accuracy and efficiency when validated against manual measurements, confirming its potential as a diagnostic and treatment planning tool for spinal disorders [38].

In addition to joint, ligament, and bone evaluation, AI-enhanced 3D CT reconstruction has also been applied in the diagnosis and surgical planning of benign bone tumors, such as osteochondromas. The utilization of 3D reconstruction in this context led to better identification of osteochondroma morphology and position, as evidenced by high radiologist agreement, and resulted in surgical planning accuracy improvements by 25% over conventional CT methods [39].

Furthermore, musculoskeletal imaging is also one of the areas where the use of robotics has found a unique application in medicine. A new technique integrated arthroscopic-assisted fusion with robot-assisted fixation to treat scaphoid non-union advanced collapse, which led to precise screw placement on the first attempt [40]. These new technologies demonstrate improvements in both surgical precision and patient outcomes.

Cardiothoracic Imaging

AI-driven 3D reconstruction has been shown to improve visualization, diagnostic accuracy, and treatment planning in a wide variety of cardiovascular and pulmonary pathologies. Deep learning methods for denoising cardiac images produce better image quality for visualizing perfusion defects compared to traditional techniques, which may help reduce the higher doses of radiation that single-photon emission computed tomography myocardial perfusion imaging (MPI) often requires [41]. A machine learning algorithm has also been developed to estimate MPI scores from 3D stereoscopic images, achieving 79.6% concordance with expert assessments and offering potential for improved MPI analysis [42]. 3D reconstruction has also been very promising in the field of structural heart disease, particularly in cardiac valve assessment. One study demonstrated that the aortic valve area in aortic stenosis can be accurately measured using 3D transesophageal echocardiography, providing a non-invasive alternative to standard methods [43].

**Figure 4:**
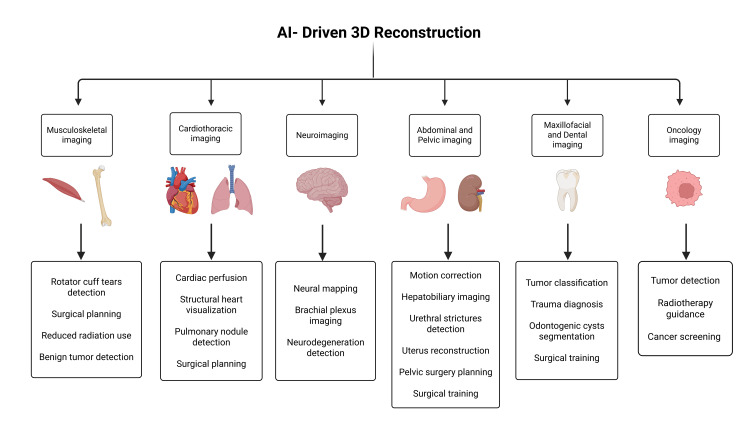
Clinical applications of AI-driven 3D reconstruction across imaging specialties. This figure was created using the software BioRender by the authors. AI = artificial intelligence; 3D = three-dimensional

Additionally, deep learning algorithms have been assessed for enhancing intracardiac echocardiography (ICE) for left atrium imaging during atrial fibrillation catheter ablation. While ICE provides real-time imaging, its lower resolution and narrower field of view limit direct anatomical equivalence with CT (Figure 4). AI-enhanced ICE has been shown to achieve moderate to high correlation with CT measurements (r = 0.52-0.75), with an average post-processing reconstruction time of 65 seconds [44].

Multiple studies have also demonstrated enhanced pulmonary procedures and diagnostics through the integration of AI with 3D reconstruction. A non-contrast CT 3D reconstruction algorithm of the pulmonary parenchyma allowed for clear and detailed visualization of pulmonary anatomy and segmentectomy surgical planning without the use of contrast [8]. Deep learning reconstruction techniques have also been applied to improve image quality for low-dose chest CT scans, making screening practices safer by reducing radiation dose without compromising diagnostic capabilities [45]. Pulmonary nodule detection has similarly been improved through AI in pulmonary MRI by increasing both the speed and sensitivity for suspicious finding identification [46].

Finally, AI-driven 3D reconstruction systems have been shown to improve preoperative planning for challenging lung surgeries. One study demonstrated improved anatomical variant identification accuracy by 8%, error reduction by 41%, and improved procedure selection accuracy by 8% with 35% fewer errors [18].

Neuroimaging

AI for 3D reconstruction has made major advances in the field of neuroimaging by improving MRI and CT brain mapping, surgical planning techniques, and lesion characterization. Deep learning reconstruction methods, such as CNNs and generative adversarial networks, have enhanced the accuracy and speed of brain image reconstruction, even from undersampled or noisy datasets [47-49].

One study demonstrated that a deep learning-based reconstruction method (DLRecon) significantly enhanced image quality and diagnostic confidence in evaluating the brachial and lumbosacral plexus compared to standard-of-care reconstruction. DLRecon also resulted in a higher contrast-to-noise ratio and signal-to-noise ratio, improving the visibility of nerve branches and pathology [50]. Another study designed a deep learning model for diagnosing Alzheimer’s disease from brain MRI scans with minimal annotations, achieving a sensitivity of 99.69% and outperforming other approaches [14].

Abdominal and Pelvic Imaging

AI-driven 3D reconstruction techniques have been applied across abdominal and pelvic imaging. A model-based deep learning framework with a folded image training strategy was proposed to increase the reconstruction quality of abdominal 3D T1-MRI [51]. Motion artifacts in abdominal imaging have also been addressed with a CNN-based image registration framework, enabling respiratory motion correction for free-breathing 3D MRI [52]. For hepatobiliary evaluation, deep learning can accelerate 3D magnetic resonance cholangiopancreatography while preserving image resolution at shorter acquisition times [53].

In genitourinary imaging, AI-assisted 3D ultrasound has shown high correlation with intraoperative measures for assessing urethral strictures [36]. In gynecology, an AI-assisted framework for autonomous uterus reconstruction using 3D SPACE MRI and iterative denoising improved soft tissue visualization [54].

AI-enhanced 3D reconstruction has also improved surgical precision and safety. An AI-based Hyper Accuracy Three-Dimensional model enabled precise tumor removal and selective vessel clamping during robotic kidney surgery, while maintaining patient safety with no surgical complications and only 14 minutes of warm ischemia time [11]. 3D anatomical models have also been utilized in surgical training for intestinal anastomosis and as educational tools in pancreatic surgery, demonstrating value in evaluating performance and improving clinical skills [55,56].

Maxillofacial and Dental Imaging

Deep learning models are increasingly applied to CT reconstruction, bone segmentation, and virtual planning workflows in oral and maxillofacial surgery (Figure 4). Nasal bone fractures have been identified accurately and automatically in CT data using deep learning approaches, while 3D model-based occlusal plane analysis has been used to predict mandibular positional changes [24,57].

AI applications to maxillofacial radiology education are also being studied, with 3D printed and virtual simulation systems used to improve preclinical oral and maxillofacial surgery training in dental students [58].

Oncology

AI-powered 3D reconstruction is transforming oncologic diagnosis, treatment planning, and therapeutic procedures. Synchrotron radiation phase-contrast CT has been used to study tumor tissue microstructures, developing a method for non-destructive 3D visualization and quantitative tumor evolution classification [59].

In lung cancer, photon-counting-detector CT combined with deep learning significantly improved image quality and diagnostic confidence [60]. Virtual 3D thin-section CT was applied to measure early-stage lung adenocarcinoma solid components, aiding nodule characterization and staging [61]. Deep learning-enhanced digital tomosynthesis has also improved tumor localization for radiotherapy-guided lung cancer treatment, enabling more accurate and efficient planning [62].

Applications extend to gastrointestinal cancers as well. AI-based segmentation of esophageal cancer and metastatic lymph nodes on CT angiography improved the delineation of tumor margins and surrounding structures [63]. Deep learning reconstruction of T2-weighted MRI improved image quality and diagnostic confidence in rectal cancer evaluation [64].

In prostate cancer imaging, deep learning has been applied to improve MRI analysis by correlating model outputs with radiologist and histopathologist evaluations. Reported diagnostic performance included an area under the curve of 0.90, supporting its potential role in prostate cancer detection [65].

Breast cancer imaging has also benefited from AI and 3D reconstruction. Deep learning has enabled volumetric breast density estimation from 3D tomosynthesis [66], graph attention networks with 3D mesh reconstructions from ultrasound images for automated diagnosis [67], and low-cost diffuse optical mammography systems validated for improved cancer detection and accessibility [68]. Collectively, these studies demonstrate that AI can improve early localization and characterization of cancer, though these tools are likely to complement rather than replace existing imaging modalities (Figure 4).

Challenges, limitations, and frontiers in AI 3D reconstruction

Although many studies indicate the promise of enhanced surgical outcomes and increased surgical efficiency, there are some limitations that need further improvement.

Studies in 3D reconstruction showed that algorithms in scenarios where anatomical variants were included have yet to be assessed, as these were very uncommon. Due to limitations inherent to some of the study designs, they may not adequately reflect the nuances of real-world clinical practice, such as order of data analysis, clinician workload, team collaboration, and real-life diagnostic errors; the improvements seen in our study were more modest than expected from a clinical standpoint [18].

In clinical settings, the optimal margin for surgery is more closely linked to the pathological margin and local recurrence rate, whereas in the studies, the choice of surgical procedure was guided by ensuring a resection margin at least equal to the nodule’s diameter on chest CT alone, which warrants further investigation [18]. Including additional landmarks and more detailed relative coordinates as calibration data for the 3D environment is anticipated to enhance the accuracy of 3D shape reconstruction [69].

Reproducibility and interpretability remain key challenges for AI-based 3D reconstruction. Many deep learning models operate as “black boxes,” making it difficult for clinicians to evaluate decision pathways or identify sources of segmentation errors. Overfitting can occur when algorithms are trained on limited or homogeneous datasets, leading to reduced performance when applied across different scanners, pathology types, or imaging protocols. Increasing algorithm transparency, using explainable AI methods, and validating performance through multi-center datasets will be essential to support reliable deployment in clinical practice.

Moreover, reviews on 3D reconstruction noted that CT scans tailored for human viewing may not serve as the most suitable input for deep learning, as AI focuses purely on pattern recognition within the imaging data, without considering the visual elements intended for human interpretation in tasks such as segmentation and surgical planning. Moreover, the variability in the data used to train neural networks makes it difficult to form broad, generalizable conclusions. To allow more effective comparisons between different neural network approaches, standardized benchmark datasets are needed [4].

Another major limitation is generalizability across diverse healthcare environments. Model performance can vary significantly depending on imaging acquisition parameters, scanner manufacturers, patient demographics, and disease prevalence. Many algorithms are validated on single-center datasets that may not represent broader clinical populations, which can lead to reduced reliability when deployed in different institutions or geographic regions. Expanding multi-center collaborations and incorporating heterogeneous datasets will be essential to ensure AI-driven 3D reconstruction systems perform consistently in real-world practice.

Furthermore, the early lung adenocarcinoma detection with 3D image was a retrospective, single-center study. More studies with a wider dataset should be utilized for future research. Although conventional CT imaging from various systems was procured, trials of CT systems from all manufacturers were not feasible. Further, unnecessary surgeries may occur due to the virtual error margin, as the study primarily focused on the solid size of lung cancers and did not assess edge features such as spiculation. Expanding the training dataset could help improve the AI algorithm’s performance in this area [61].

In osseous studies, standard MRI sequences tend to overestimate bone lesions, such as erosions or cysts in rheumatic conditions, because of fluid signals, unlike MR sequences such as ultra-short echo time or zero echo time, which mimic CT and provide a more accurate assessment [70].

Greater focus should be placed on improving the integration and collaboration between 3D reconstruction techniques and healthcare professionals, considering the complexity of real-world clinical decision-making. Biases may be introduced by using multi-view radiograph inputs due to style transfer problems. Consequently, future research should prioritize gathering real-world biplanar radiographs as a crucial step [69].

Lastly, one of the key challenges of the proposed schemes is their high computational cost and resources, which may limit their practicality and scalability in real-world applications [71,72].

**Figure 5:**
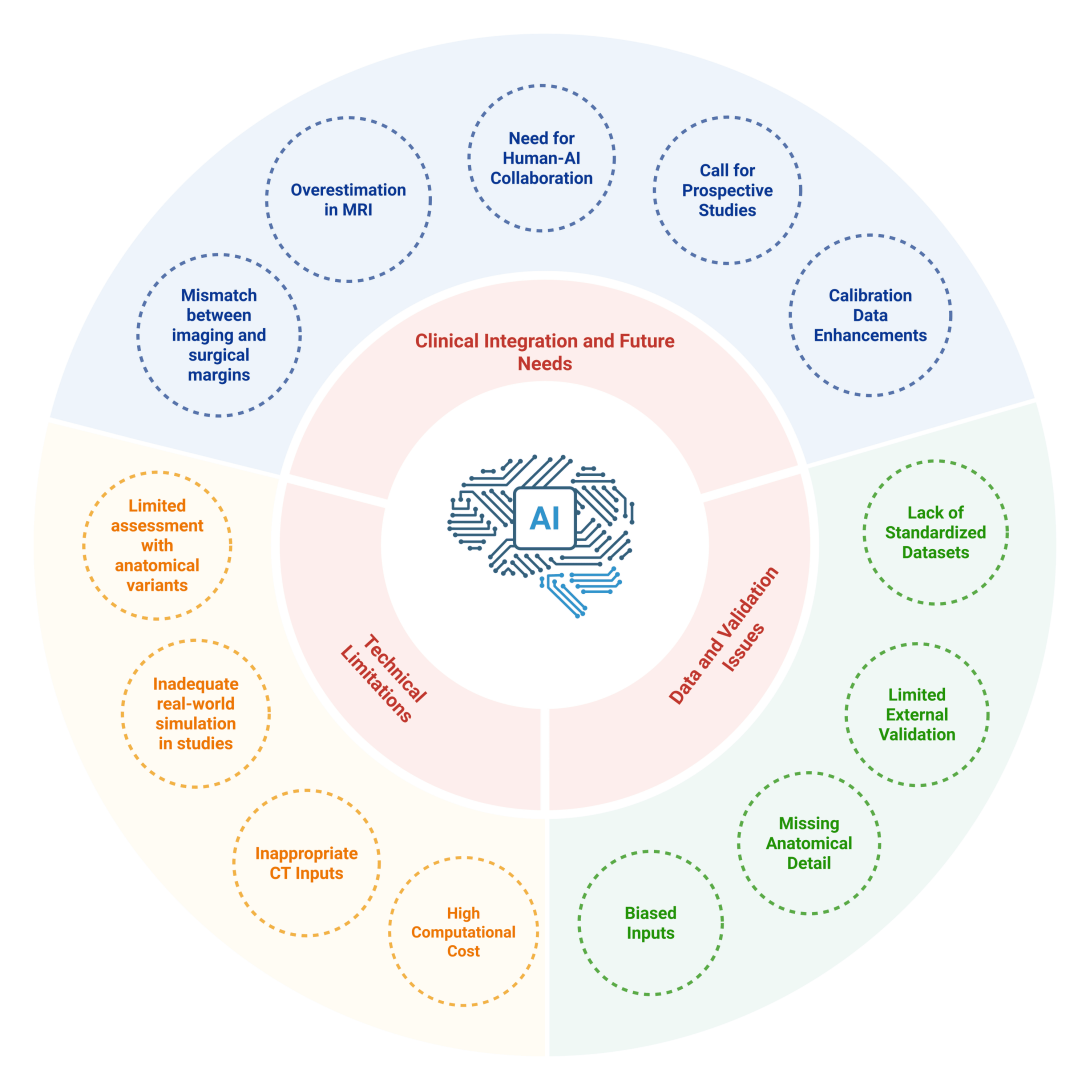
Current limitations and future innovations in AI-based 3D reconstruction. This figure was created using the software BioRender by the authors. AI = artificial intelligence; 3D = three-dimensional

Additional research, particularly prospective studies that incorporate perioperative patient outcomes, is essential to confirm the clinical utility of the AI-3D system. These critical aspects lie outside the scope of current studies and should be explored in future investigations. The current limitations and future innovations in AI-based 3D reconstruction have been summarized in Figure 5.

Ethical challenges and pathways for future integration

Developments and innovations in radiology are reshaping how clinicians visualize anatomy and interpret complex imaging data. One such milestone is AI-driven 3D reconstruction techniques. On the one hand, AI technologies have promising potential in improving diagnostic accuracy, surgical planning, and patient outcomes; however, they also raise important ethical and privacy-related questions that cannot be overlooked.

AI models are fine-tuned for reading scans with massive volumes of old and new imaging data, and that raises considerable concern about ethical data extraction [73]. The collection and use of these large volumes of patient medical data increases the risk of unauthorized access, confidentiality breaches, and potential data leaks. Even though data are anonymized, the current method of deidentifying data does not adequately protect data and does not safeguard personal information [74]. Further, with the advancement in data linkage and identification techniques, there is a risk of re-identifying and tracing back the images to the patient [74,75]. This challenges our traditional notion of informed consent and privacy, as most of the dataset was not originally collected with AI development in mind, thus patients may not have consented to their images being reused the way it is [74].

Most AI systems are owned and operated by private entities, which allows them access to important patient data, raising questions about how this information is used and protected. Thus, to address these risks, there is a strong need for better oversight, structural safeguards, and updated regulations that prioritize patient consent and more robust deidentification and data protection techniques [73].

Risk and opportunity assessment are important when an organization plans to implement AI in its systems. As AI becomes more integrated in radiology, it also shines light on cybersecurity threats and the need to recognize them and ensure safe integration. Implementing AI in radiology presents new digital security challenges in safeguarding patient confidentiality and ensuring data accuracy, completeness, and consistency, challenges that often extend beyond just image interpretation [76].

Therefore, looking ahead, integration of AI in healthcare clearly needs an ethical framework and guidelines for how AI should be developed and deployed. This includes not only compliance with existing regulations such as the General Data Protection Regulation (GDPR) and the Health Insurance Portability and Accountability (HIPAA) but also broader principles such as transparency, accountability, and respect for patient autonomy. These ethical considerations should be the framework for building any AI system for healthcare. If that is maintained, there are many exciting opportunities for this AI system for international collaborations that can help with building large, ethically sourced, and diverse datasets to train and develop more robust models [77].

**Figure 6:**
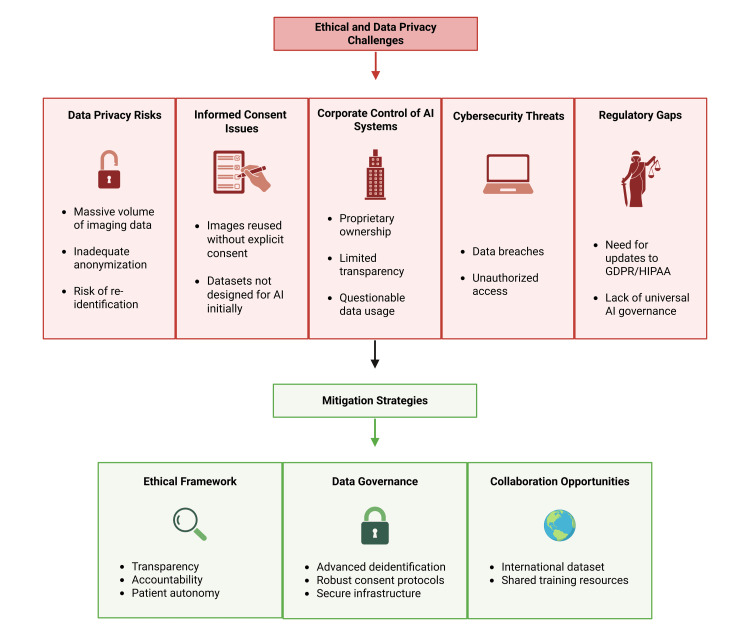
Ethical and regulatory considerations in AI imaging integration. This figure was created using the software BioRender by the authors. AI = artificial intelligence; GDPR = General Data Protection Regulation; HIPAA = Health Insurance Portability and Accountability

Overall, while AI integration in radiology holds incredible potential in transforming the field, it also presents a set of ethical and privacy challenges that must be addressed head-on (Figure 6). By developing inclusive, transparent, and secure systems and by involving clinicians, patients, and regulators in decision-making and consent, we can ensure that these tools are used responsibly and for the benefit of all.

Data privacy safeguards and regulatory oversight

Despite the increased utilization of AI in radiology and its potential future, this innovation also carries limitations and high risks. Several challenges should be addressed before AI becomes more widely accepted in the field. Many of the AI applications are developed and owned by private entities and big corporations, which means they have a major role in obtaining, using, and protecting patient privacy data and health information [73]. This raises a significant privacy concern relating to data security and protection.

There are several ethical issues with access and control of data, which have resulted in poor protection of patients’ privacy information. This highlights the urgent need for regulatory bodies to develop ethical and data privacy guidance to promote legal and safe use of AI and enhance its application. Therefore, appropriate regulations and policies should be implemented to protect data privacy and place greater emphasis on patient consent. There is also a need to use more advanced tools for data anonymization and security to enhance the protection of health-related information [19].

Future directions and clinical opportunities

The rapid technical advancement in medical AI has the potential to improve patient outcomes and transform healthcare. The use of AI in diagnostic radiology holds a promising future, with many applications being reported in the literature. AI may provide more efficient and accurate results, allowing clinicians to spend less time on daily routine tasks and more time with patients. It has the capacity to shorten the diagnostic time as well as the amount of effort required to complete the task. It can also enhance precision and improve visualization of the images [16].

A common challenge of AI tools in radiology is generalizability and reproducibility of the data [78]. The resulting outcome for most real-world AI applications lacks consistency when compared to clinical research due to limited databases. It is crucial that when developing an AI application in radiology, a high-quality database for algorithm training must be used that represents the general population [79]. Future investigators should develop better study designs and data-sharing protocols between institutions to obtain a high-quality database with different clinical settings, geographic locations, genders, ethnicities, and ages.

Moreover, there is a knowledge gap among radiologists about the benefit of utilizing AI in their practice [78]. Future medical education should incorporate AI in its curriculum to help reduce the knowledge gap and the lack of trust by physicians [16].

In addition, future researchers should investigate the utilization of AI in radiology to develop a more personalized treatment plan and further predict disease progression and monitoring. Moreover, there is a need to evaluate the use of 3D models from radiological scans in preoperative planning to optimize the outcome of the procedures and minimize complications. There is also a promising opportunity with the use of AI 3D imaging in creating personalized medical devices and custom prosthetics with a better custom-fitted design, comfort, and functionality [16].

## Conclusions

With the rapid advancement of AI in the medical field, AI in radiology holds a strong clinical trajectory, with several applications already being utilized in practice. This study summarized current opportunities in radiology, including applications from data collection and 3D reconstruction to image evaluation and analysis. However, the current evidence base is limited by single-center designs, heterogeneous evaluation metrics, and variability in clinical validation methods. To strengthen the evidence base, future research should focus on multicenter and longitudinal studies assessing whether AI-assisted 3D models improve diagnostic accuracy, surgical planning, and patient outcomes, alongside robust evaluation of data governance, privacy, and integration within public healthcare systems. Standardized benchmarking and transparent model reporting will be essential for reliable everyday adoption.
